# Linkage analysis coupled with exome sequencing identified defects in gene ‘X’ causing premature ovarian insufficiency

**DOI:** 10.1186/1471-2164-15-S2-P6

**Published:** 2014-04-02

**Authors:** Sulman Basit

**Affiliations:** 1 Center for Genetics and Inherited Diseases, Taibah University Madinah Al-Munawara, Kingdom of Saudi Arabia

## Background

Premature ovarian insufficiency (POI) is defined as a primary ovarian defect characterized by absent menarche (primary amenorrhea) or premature depletion of ovarian follicles before the age of 40 (secondary amenorrhea) with hypergonadotropism and hypoestrogenism. POI results in infertility and lifelong steroid deficiency, and is potentially associated with accelerated health risks such as cardiovascular and neurodegenerative disorders and osteoporosis.

## Materials and methods

A large consanguineous Saudi family with three female affected with POI was investigated. All samples including 3 affected and 6 unaffected underwent whole genome SNP genotyping using Affymetric 250K array. Linkage analysis was carried out using HomozygosityMapper and Allegro software. Candidate gene sequencing was performed using ABI3500 genetic analyzer. Whole exome was sequenced in three affected and one normal individual using life technologies Ion Proton sequencer.

## Results

Linkage analysis mapped the disease phenotype to long arm of chromosome 20. Sequence data analysis of potential candidate genes failed to detect any pathogenic variant. Exome sequencing data analysis identified a deletion mutation in gene ‘X’ on long arm of chromosome 20. This mutation is perfectly segregating with the disease phenotype in pedigree.

## Conclusions

We identified a novel gene responsible for POI in Saudi Arabian family. Our findings extend the body of evidence that supports the importance of gene ‘X’ in the development of ovary and ovarian reserves.
